# Early Life Beef Consumption Patterns Are Related to Cognitive Outcomes at 1–5 Years of Age: An Exploratory Study

**DOI:** 10.3390/nu14214497

**Published:** 2022-10-26

**Authors:** Victoria C. Wilk, Michelle K. McGuire, Annie J. Roe

**Affiliations:** Margaret Ritchie School of Family and Consumer Sciences, College of Agricultural and Life Sciences, University of Idaho, Moscow, ID 83844, USA

**Keywords:** beef, iron, zinc, choline, protein, infant, cognition, inhibitory control, attention

## Abstract

Protein, iron, zinc, and choline affect early brain development and are found in beef. The aims of this study were to describe (1) early feeding practices related to introduction of beef in the rural US west (Idaho); (2) parental perceptions of beef as a first food, and (3) associations between early beef consumption and child cognition at 1–5 years. A total of 61 children and their parents were enrolled. Parents completed a survey and a food frequency questionnaire to assess perceptions of beef and early feeding practices along with their child’s dietary intake at 6–12 months. Children’s cognitive function was assessed using the Bayley-4 Scales of Infant and Toddler Development (12–35 months) and the NIH Toolbox for Assessment of Neurological and Behavioral Function (NIHTB) (3–5 years). Parents introduced beef at 7.79 ± 2.65 months of age, primarily so that their children could eat what the family was eating. Higher intake of beef (r = 0.41, *p* = 0.02), zinc (r = 0.45, *p* = 0.01), and choline (r = 0.39, *p* = 0.03) at 6–12 months was associated with better attention and inhibitory control at 3–5 years of age. These findings support the role of beef as an early food for cognitive development, although controlled dietary intervention studies are needed.

## 1. Introduction

Adequate nutrition in utero and the first two years of life is critical for optimal brain development and cognitive function later in life [1]. Suboptimal intake of key nutrients in the first 1000 days may result in cognitive deficits that cannot be reversed through improved intake later in life [2]. The Dietary Guidelines for Americans now include recommendations for children from birth to 2 years of age and highlight the importance of early nutritional needs [3]. A diet composed exclusively of human milk is adequate for most infants during the first 6 months of life [4,5]. Breastfeeding benefits infants by reducing risk of infections [6,7], enhancing neurodevelopment [8,9], and providing a plethora of important nutrients essential for healthy development [10]. Human milk is a nutrient-rich biological system that can sustain an infant for the first 6 months of life, but eventually children require nutrients beyond those provided in breastmilk. To supplement infant feeding, the Dietary Guidelines for Americans and the American Academy of Pediatrics recommend introducing foods (known also as complementary foods) other than human milk and formula at approximately 6 months of age [3,11].

While children obtain diverse nutrition from complementary feeding, nutrients of concern remain. These are nutrients that are not being consumed at recommended levels yet are vital to development. Protein, iron, zinc, and choline are all nutrients that affect early life brain development [12,13]. These nutrients, along with vitamin D and potassium, were noted as nutrients of concern and under-consumption among infants ages 6 to 11 months by the most recent U.S. Dietary Guidelines for Americans Advisory Committee [14]. Iron and zinc deficiency are common in growing children, with mean iron intake among infants in decline [15,16].

Some parents choose to feed their infants fortified infant formulas to prevent nutrient deficiencies, while others incorporate everyday foods in the form of mashing, puree, or liquids into their child’s diet. The World Health Organization (WHO) recommends that parents feed their children animal-based products to meet the nutritional needs of children during the first few years of their life [17]. Animal-based products have been shown to reduce iron [18] and zinc [19] deficiencies. Beef, in particular, has been found to provide high levels of both iron and zinc and is a nutrient-dense option for infants as a first food [20,21]. Beef is also high in choline and vitamin B12, both of which play roles in neurodevelopment and cognition [22]. Despite studies showing beef and other meat as a good source of nutrients important for cognitive development, few studies have assessed relationships between early beef intake and child cognition. A systematic review in 2019 reported on only eight studies and concluded inconsistent results, with only one study reporting improved cognitive function with increased intake of beef [23]. This study was not conducted in young children but rather in young women [24].

Research on how parental perceptions of beef as a first food and infant feeding practices influence nutritional status of infants and toddlers is limited. The majority of the research focuses on women with low resources or from developing countries or focuses on weight gain as an outcome [25,26]. A study conducted in August of 2020 by the National Cattlemen’s Beef Association investigated the feeding habits of parents with a child who is 6 to 24 months old. The study explored elements considered when choosing food, timing of beef introduction, and knowledge of beef nutritional value. Results suggested that over half of surveyed parents believed that nutritional value was the top benefit of beef, yet only 30% of parents fed their children beef before year one of life [27].

The National Cattlemen’s Beef Association’s study was very useful for dietitians and nutritionists to understand parents’ perceptions of beef throughout the entire nation, but no study has been conducted in the rural west, such as Idaho. According to the U.S. Department of Agriculture (USDA), Idaho had 2.4 million cattle in 2017, outnumbering the human population by over 700,000. The USDA also ranked Idaho 12th among all states in cattle inventory and 11th in cattle sales [28]. Considering that Idaho’s beef economy is substantially larger than most states, information needs to be collected to understand the impact of early beef intake on the development and health of Idaho infants and to investigate the perceptions of beef as a first food in infant feeding. This study aimed to describe (1) early feeding practices related to introduction of beef in the rural US west (Idaho); (2) parental perceptions of beef as a first food; and (3) associations between early dietary beef, protein, iron, zinc, and choline intake and child cognition at 1–5 years of age.

## 2. Materials and Methods

### 2.1. Study Design

This observational study consisted of a survey assessing perceptions and practices of early beef and feeding practices, a cognitive assessment, and a retrospective food frequency questionnaire. Data were collected from January through June 2022. Idaho parents/caregivers were recruited to participate in this study with their children, all of whom were 1–5 years old. Subjects were recruited using voluntary sampling methods including convenience sampling, which included posting hard-copy and electronic flyers, and snowball sampling, which included asking enrolled subjects to share the recruitment flyer with interested friends or family [29]. Posted flyers gave individuals access to a screening form prior to the study to determine if they met eligibility criteria. Individuals were eligible if they had a child between the ages of 1 and 5 years, were the primary person responsible for feeding the child between ages 6 and 12 months, ≥18 years, and currently resided in Idaho. Once determined eligible, parents were sent an electronic consent form and the parent/child dyad was enrolled.

Once enrolled, parents were sent a 20-min electronic survey administered using Qualtrics software through the University of Idaho (Qualtrics^®^ Software Company Provo, Utah, USA, January 2022). This survey gathered information on perceptions of beef, infant feeding practices, and demographic information. Upon completion of the online survey, parents were contacted to schedule an in-person visit for themselves and their child. At this session, the child completed a cognitive assessment, and height and weight were measured. The parent participated in a researcher-led interview to complete a retrospective food frequency questionnaire. Upon completion of all parts of the study, subject pairs were provided an electronic gift card to compensate for their time and effort in the study. All procedures were approved by the University of Idaho Institutional Review board, and informed consent and assent (as appropriate for age) were obtained.

### 2.2. Perceptions and Practices Survey

The perceptions and practices survey consisted of five sections: (1) child information; (2) feeding practices and preferences; (3) food purchasing and preparation; (4) sources of information regarding early life feeding; and (5) demographics. The survey took approximately 20 min.

Several questions on the survey were created based on questions asked in the National Cattlemen’s Beef Association Early Years Survey conducted in 2020, which assessed beef feeding practices and perceptions in the U.S. [27]. Questions were also created based on conversations with the National Cattlemen’s Beef Association, as well as the personal expertise of the research team. The survey was reviewed by two subject-matter experts outside of the research team and revised based on their feedback. A pilot survey was sent to 37 Idaho parents/caregivers before the main study began, which allowed researchers to optimize survey formatting, content, and phrasing. The survey used a variety of questions, both fill-in format and selection format, and Likert-scaling to assess parents’ perceptions of beef and their use of beef in infant feeding. To help counteract bias and minimize testing threat with subjects guessing the study was focused on beef feeding practices, the survey also asked questions about other types of meat, such as pork, fish, and chicken.

### 2.3. Cognitive Assessments

Cognitive assessments were performed between the hours of 8:00 a.m. and 4:00 p.m. Researchers explained the assessments in simple terms to the children and obtained assent. Children were offered a snack before testing to ensure that hunger had minimal effects on scores [30]. Accommodations were made upon participant request, such as having shoes off, changing locations in the room, or having a beverage or snack nearby, in order to minimize discomfort or distractions [30].

The cognitive subtest of the Bayley-4 Scales of Infant and Toddler Development (Bayley-4) was used to assess cognition in children 12 to 35 months of age [31]. This test battery includes items to assess visual performance, attention, memory, sensorimotor, exploration and manipulation, and concept formation. A trained researcher conducted the assessment in a quiet room with a parent present while recording behavioral responses on an iPad. Approximately halfway through the assessment, the researcher gave subjects a break by measuring their height and weight before continuing. The assessment length varied depending on the age and abilities of the child. The Standard score, or overall score, was used to interpret results. This composite score has the highest internal consistency reliability of the scores generated and has a mean of 100 and standard deviation of 15 [32].

The fluid cognition assessment battery of the National Institute of Health Toolbox for Assessment of Neurological and Behavioral Function (NIHTB-CB) was used to assess cognition in children 3–5 years of age [33]. The assessments included in this battery are summarized in Table 1. A trained researcher conducted the assessment in a quiet room using an iPad that was placed within a comfortable reach of the seated participant on a table. After two of the five assessments had been completed, researchers gave subjects a break by measuring their height and weight before continuing with the last three games. The assessment took approximately 50 min, and subjects were able to take a break whenever requested. Parents were allowed to be in the room with the child if needed, although they were asked to not interfere with testing.

The five assessments within the NIHTB-CB were both individually scored by the application and used to generate a fluid cognition composite score. The fully corrected T-score was used to interpret results, which compares the score of the participant to normative national averages, while adjusting for demographic information, including age, gender, race/ethnicity, and parent educational attainment (the score has a mean of 50 and a standard deviation of 10).

### 2.4. Food Frequency Questionnaire

A retrospective food frequency questionnaire was developed to estimate dietary intake at 6–12 months of age. The food frequency questionnaire was developed based on a previously validated questionnaire designed to estimate dietary intake during the first two years of life [34]. Since the previous questionnaire was developed using foods common in rural Mexico, only the format and organization were used. Many foods were replaced with ones that were more common in Idaho familial households. Foods were chosen based on other U.S. based infant and toddler dietary intake reports [35,36].

The food frequency questionnaire was administered via an interview format. The same researcher conducted all food frequency questionnaire interviews. Six categories of foods were included in the questionnaire: liquids, dairy, cereals and starches, meats, sweets, and other (which included fruits, vegetables, lentils, nuts, and supplements). Parents were asked how often a food/liquid was consumed by the child (per day, week, or month) and how much of it they would eat in one sitting, during months 6–12 of the child’s life. If the amount/frequency of the food varied over the span of the 6 months, parents were asked to average the amount/frequency. At the end of each food category, parents were asked if there were any foods not covered in the food frequency questionnaire that they would like to add, and additional foods were added according to each individual participant. Subjects were encouraged to send any future email or text updates about foods they may have forgotten during the interview. Items that represented standard measurement were provided to aid subjects in choosing accurate measurements. The questionnaire lasted approximately 20 min and took place at the Ramsay Research Unit on the University of Idaho campus or via Zoom, if it was more convenient for the participant. Once food frequency questionnaires were conducted, each food item and daily intake amount was entered into the ESHA Research Food Processor, a nutrition analysis software [37]. If food were eaten weekly rather than daily, the amount of food per week was divided by 7 to estimate average daily intake. The ESHA Research Food Processor was then used to estimate the average daily intake of beef, protein, iron, zinc, and choline.

### 2.5. Data Analysis

Data analysis was conducted using SAS^®^ software, version 9.4 (copyright © 2002–2012, SAS Institute Inc., Cary, NC, USA). Descriptive statistics were used to summarize characteristics of the sample population, including demographic data, cognitive scores, dietary intake data, and data from the perceptions and practices survey. Numerical variables were reported as mean and standard deviation, and categorical variables were reported as frequencies.

Dietary intake data and cognitive scores were evaluated for characteristics of normality by visual evaluation. Outliers for dietary intake (beef, protein, iron, zinc, choline) and cognitive scores were identified if they were 1.5 times the interquartile range greater than the third quartile, or 1.5 times the interquartile range less than the first quartile. Relationships between cognitive scores and dietary intake were assessed via Spearman rank correlation. Missing values were omitted from the final data analysis.

## 3. Results

### 3.1. Subject Characteristics

**Figure 1:**
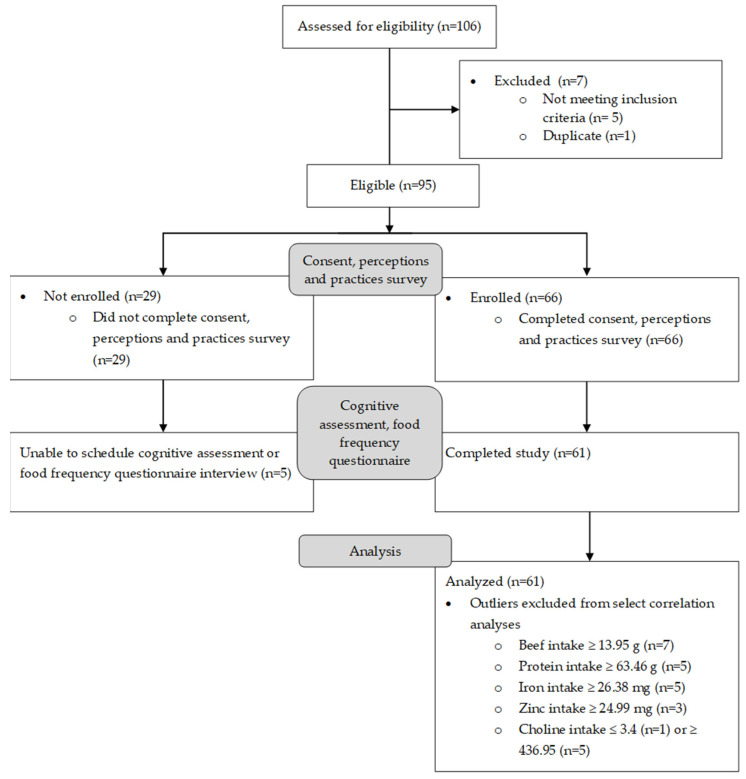
Consort diagram of eligible, enrolled, and analyzed subjects.

Figure 1 shows the flow of subjects from recruitment to final analysis. A total of 106 individuals completed the screening form and were contacted to enroll in the study. Of these, 5 were ineligible due to state of residency, 1 was a duplicate (2 parents completed separate forms for the same child), and 1 individual did not answer phone calls or respond to attempts to contact. Of the remaining 95 eligible individuals, 29 expressed interest and were sent the link to the consent and perceptions and practices survey but did not complete these documents and so were not scheduled for the in-person assessments, 5 completed the consent and survey but were unable to complete the in-person assessments due to scheduling conflicts or illnesses, 61 individuals completed all aspects of the study. This sample size provided 80% power to detect a correlation of r = 0.353, at a significance level of 5% [38].

Subject characteristics are summarized in Table 2. In brief, the sample population was from well-educated, higher-income households. The majority were white and non-Hispanic. Parents introduced beef to their child’s diet at an average age of 7.79 ± 2.65 months of age.

### 3.2. Perceptions and Practices Survey

**Figure 2:**
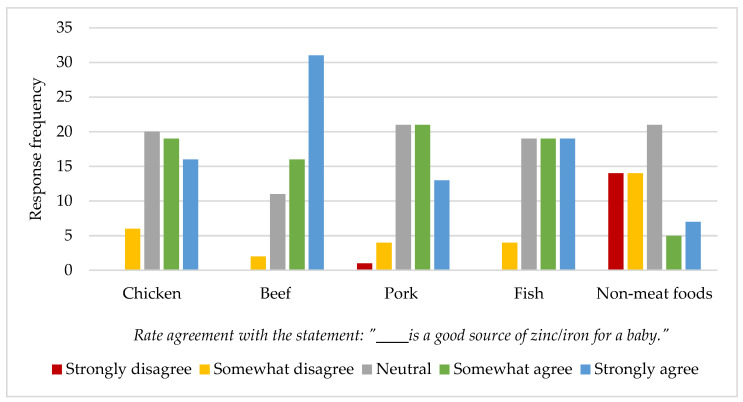
Idaho parent/caregiver’s perception of beef and other foods as a good source of zinc/iron for infants. Subjects were asked in the form of an electronic Qualtrics survey (*n* = 61).

Parents were asked to rate their agreement with the statement that beef (or chicken, pork, fish, non-meat sources) is a good source of zinc/iron for a baby. Results are summarized in Figure 2. Seventy-eight percent of respondents somewhat agreed or strongly agreed with the statement that beef is a good source of zinc/iron for a baby.

**Figure 3:**
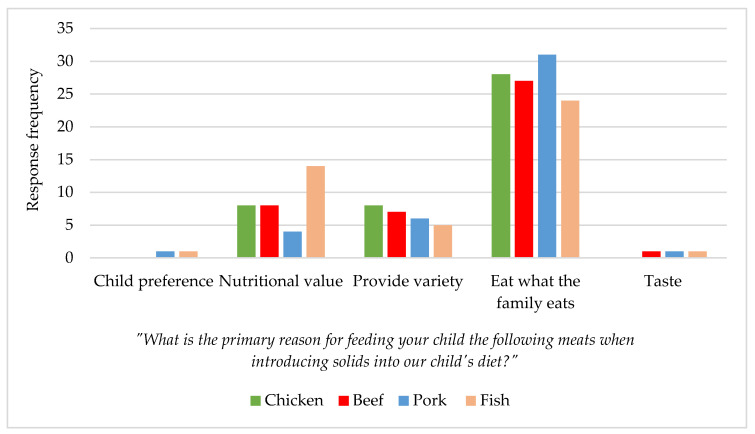
Idaho parent/caregiver primary reason for choosing meat when introducing solids. Subjects were asked in the form of an electronic Qualtrics survey (*n* = 45).

In the sample of 61 Idaho parents/caregivers, 26% of respondents did not introduce meats into their child’s diet at 6–12 months of age. Of those who did introduce meat, the most commonly chosen reason for introducing any meat into their child’s diet was so that they would eat what the rest of the family eats (see Figure 3). Sixty-three percent of respondents indicated introducing beef so that their child would eat what the rest of the family eats.

**Figure 4:**
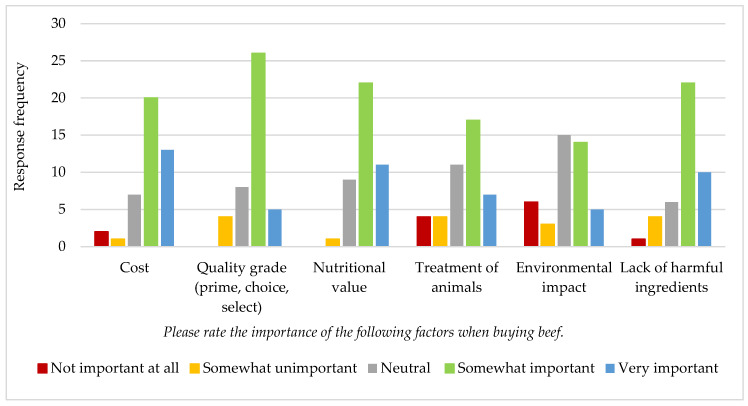
Importance of various factors to Idaho parents when buying beef (*n* = 43).

Parents were asked to rate the importance of different factors when buying beef. The results are summarized in Figure 4. Over 70% of respondents reported cost, quality, nutrition, and lack of harmful ingredients as somewhat or very important.

### 3.3. Retrospective Dietary Intake

Child dietary intake at age 6–12 months was estimated based on the retrospective food frequency questionnaire. Results for dietary intake of beef, protein, iron, zinc, and choline are summarized in Table 3 along with the corresponding Recommended Dietary Allowance or Adequate Intake [39,40,41].

Relationships between average beef intake and intake of protein, iron, zinc, and choline were assessed by Spearman rank correlation. Higher intake of beef was significantly related to higher intake of protein (r = 0.31, *p* = 0.01), zinc (r = 0.31, *p* = 0.02), and choline (r = 0.37, *p* = 0.004), but not iron (r = 0.15, *p* = 0.26). The relationships with zinc and choline remained significant when outliers were removed, but the relationship with protein was attenuated.

Using the interquartile range method, outliers were identified as beef intake ≥ 13.93 g (*n* = 7), protein intake ≥ 63.46 g (*n* = 5), iron intake ≥ 26.38 mg (*n* = 5), zinc intake ≥ 24.99 mg (*n* = 3), and choline intake ≤ 3.4 (*n* = 1) or ≥ 436.95 (*n* = 5).

### 3.4. Cognitive Assessment

Scores on cognitive assessment tests are summarized in Table 4. Children aged 12–35 months of age received a Standard score from the Bayley-4 assessment (the score has a mean of 100 and standard deviation of 15). Children aged 3–5 years of age received individual scores for each of the 5 assessments in the NIHTB-CB, as well as a composite score of overall fluid cognition. The fully corrected T-scores presented in Table 4 are adjusted for age, sex, race/ethnicity, and parental education attainment (the score has a mean of 50 and standard deviation of 10). If subjects were unable to complete the tutorial section of any test, they did not receive a score for that assessment. If a subject did not receive a score on any of the 5 subtests, a Fluid Cognition Composite score was not calculated. This resulted in different sample sizes for each cognitive score. Using the interquartile range method, outliers were identified as a Standard score ≤ 65 (*n* = 1) and a Picture Sequence Memory score ≥ 78 (*n* = 1). There were no outliers for any of the other cognitive scores.

### 3.5. Relationships between Diet and Cognition

Relationships between dietary intake of beef, protein, iron, zinc, and choline at 6–12 months of age and cognitive scores at age 1–5 years of age are summarized in Table 5. Higher average daily intake of beef, protein, zinc, and choline were significantly correlated with higher scores on the Flanker Inhibitory Control and Attention test. The relationship with beef, zinc, and choline remained significant when removing outliers, while the relationship with protein was attenuated.

## 4. Discussion

This observational study captured descriptive data on Idaho parents’ perceptions of beef and other types of meat as a first food as well as feeding practices when their children were 6–12 months of age and assessed for relationships between early dietary intake and child cognitive scores at 1–5 years of age. Idaho parents and caregivers reported introducing beef to their children at around 7 months of age, primarily so that their children could eat what the rest of the family was eating. Children whose parents reported higher average daily beef, zinc, and choline intakes at 6–12 months of age scored higher on a test of inhibitory control and attention at 3–5 years of age.

The results of the perceptions and practices survey showed that parents typically followed the Dietary Guidelines for Americans recommendation to introduce complementary foods at the age of six months, with most exclusively breastfeeding their child before the age of six months [14]. Parents also generally followed guidelines with supplementary feedings of breastmilk/formula until at least the age of 1 year. Infant wellbeing was the focus when parents were answering questions about early feeding practices, with nutrition and infant safety being the top values when choosing first foods for their infants, and medical experts being the most common consultants to parents. Idaho parents saw beef favorably as a nutritional food but had worries about their infants chewing and choking on beef as a first food. Parents reported introducing beef when their child was 7.79 ± 2.65 months of age. This was slightly younger than when they introduced pork or fish, but slighter older than when introducing chicken. When buying beef, cost and nutritional value were both highly important for parents of infants. These results were similar to national results, which showed that parents value nutrition when choosing first foods for their infant, and are concerned about choking, chewing, and safety of food [27].

Analysis of the retrospective food frequency questionnaire did not reveal any areas of significant concern for consumption of key nutrients, protein, iron, zinc, and choline in this sample population. However, the variability in the reported intakes makes it difficult to draw clear conclusions. It is also likely that parents overestimated dietary intake, as this is a known limitation of this type of dietary recall data [42].

The Recommended Dietary Allowance (amount needed to meet the nutrition needs for 97–98% of people) of protein for infants ages 6–12 months is 11.0 g/d [41]. The reported average intake for children in our study (26.5 ± 16.7 g/d) was over twice the recommended intake, but only slightly higher than the average daily intake for children aged 6–11.9 months reported in the 2016 Feeding Infants and Toddlers (FITS) database (21 ± 0.2 g/d) [41]. Even when removing outliers, the average reported daily protein intake was high (25.6 ± 10.0 g/d). Similar trends were observed with reported daily intakes of zinc and choline. Parents recalled giving their children, aged 6–12 months, foods containing 6.0 ± 5.3 mg of zinc per day, compared to the RDA of 3 mg/d and the Tolerable Upper Intake Level (UL) of 5 mg/d [40]. When removing outliers, the average zinc intake reported was 5.0 ± 3.0 mg/d. Although above the RDA, the zinc intake reported in this study was similar to the national average of 5.8 ± 0.1 mg/d reported in the 2016 FITS database for infants 6–11.9 months [43]. Similar results were observed with dietary choline intake. Parents reported feeding their children foods containing an average of 242.1 ±166.6 mg of choline per day. This is above the Adequate Intake of 150 mg/d for children 6–12 months of age [39]. When removing outliers, the mean intake was still high, but the standard deviation was lower (208.9 ± 74.1 mg/d).

Many parents fed their children iron-fortified formulas and iron-fortified baby cereals between the ages of 6–12 months, and the average daily iron intake for infants was estimated to be 9.3 ± 8.7 mg/d. The RDA for daily iron intake in 6–11-month-old infants is 11 mg/day [40]. Average daily iron intake in this study was below the national average reported in the 2016 FITS database of 13 ± 0.2 mg/d in infants 6–11.9 months of age [43]. When removing outliers, the daily iron intake reported in this study was lower (7.5 ± 6.2 mg/d). The large standard deviation in this sample makes it difficult to draw conclusions and warrants further investigation with larger sample sizes. The variability in iron intake may be in part due to the popularity of iron-fortified foods and drinks. The high intake of iron-rich foods other than beef also may be a reason that iron intake was not associated with beef intake, despite beef being a good source of iron [44].

For children aged 12–35 months, the Standard score obtained from the Bayley-4 was categorized as Average and within the 25th–74th percentile of the national validation sample [32]. For children aged 3–5 years who completed the NIHTB-CB, the average scores obtained were all within one standard deviation of the national average with the exception of the Pattern Comparison Processing Speed score which was almost two standard deviations below the average. Estimated daily beef, protein, zinc, and choline intake were all positively correlated with scores on the Flanker Inhibitory Control and Attention test prior to outlier removal, with only the correlation with daily protein intake not remaining significant after outliers were removed. This suggests that beef, or nutrients found in beef, may play a role in attention/inhibitory control. Dietary intakes were not significantly associated with any other cognitive scores.

Finding no significant relationship between dietary intake of iron and cognitive function was unexpected due to the role of iron in early brain development [22]. There was a great deal of iron variability observed in our sample population, so the relationship may not be as clear as if it was more controlled and uniform throughout the cohort. The relationship between beef intake and cognition could be influenced more by zinc, protein, and choline than iron for this reason. Furthermore, other factors are also involved with cognitive development and may be driving these findings. Beef is also a good source of vitamin B12, which has been associated with adverse cognitive outcomes if under-consumed in children [45]. The majority of children in this study were exclusively breastfed early in life, a practice that has been associated with favorable neurodevelopment [46].

### Strengths and Limitations

The perceptions and practices survey was designed to be an exploratory tool in nature, looking at the most common selected answers and trends rather than being a determinant of significant relationships. This provided hypothesis-generating data that will help justify further research. However, due to the convenience sample recruiting around the Moscow, Idaho area, our study population was largely white, high income, and well educated. As such, we acknowledge that our data do not represent all of Idaho, nor the entire rural west, which includes even less populated areas with lower income and education levels. In the future, it would be beneficial to repeat this study on a larger scale including additional western states, to include more races, rural areas, and more variability in income and educational obtainment. In addition, while the survey collected data on parent feeding practices, other parenting practices were not assessed. Future studies should collect data on parental involvement or childcare practices in order to assess the role of such variables as mediators or covariates in the relationship between dietary intake and cognition. Better understanding the interplay between other parenting practices and dietary behaviors on cognition may be important to consider when developing interventions to improve child cognitive development.

The retrospective food frequency questionnaire allowed for a longitudinal analysis with relation to cognitive outcomes, without requiring long-term follow-up. However, because we asked parents to recall daily food intake, often years in the past, there are inaccuracies in the dietary intake data, as intakes are likely to be over- or underestimated. While early dietary intake from age 6–12 months was the focus of this study, current dietary intake of subjects may have been a confounding factor that influenced the observed relationships (or lack thereof). Because this is an observational study, cause–effect relationships cannot be made based on these data. Nonetheless, our finding of a positive association between early beef consumption and an indicator of inhibitory control and attention warrants further study.

## 5. Conclusions

Idaho parents and caregivers reported introducing beef to their children at around 7 months of age, primarily so that their child would eat what the rest of the family was eating. Children whose parents reported higher average daily beef, zinc, and choline intakes at 6–12 months of age scored higher on a test of inhibitory control and attention at 3–5 years of age. It is important to note that these correlational observations do not imply causation. These results need to be confirmed in subjects from lower socioeconomic households and of various racial and ethnic backgrounds in order to make the results generalizable to a larger population. Additionally, prospective cohort and randomized controlled trials also need to be conducted in order to provide insights into causal relationships. Despite the limitations inherent in an observational study with retrospective dietary recall, the findings of this study contribute to the evidence base supporting the role of beef as an early food for cognitive development.

## Figures and Tables

**Table 1:** NIH Toolbox Cognitive Testing Battery.

Construct	Test	Description
Executive Functioning and Attention	Flanker Inhibitory Control and Attention Test	Measures attention and inhibitory control. Participant focuses on a given stimulus while inhibiting attention to stimuli flanking it.
Working Memory	List Sorting Working Memory Test	Measures working memory. Participant recalls and sequences visually and orally presented stimuli.
Executive Function	Dimensional Change Card Sort Test	Measures cognitive flexibility and attention. Pictures are presented varying along two dimensions (e.g., shape and color). The dimension for sorting is indicated by a cue word on the screen.
Processing Speed	Pattern Comparison Processing Speed Test	Measures processing speed. Subjects discern whether two side-by-side pictures are the same or not, with 85 s to respond to as many items as possible. Items are simple to purely measure processing speed.
Episodic Memory	Picture Sequence Memory Test	Measures episodic memory. Subjects are asked to reproduce a sequence of pictures that is shown on the screen. Different practice sequences and test items for subjects of different ages.

**Table 2:** Subject characteristics. SD indicates standard deviation (*n* = 61).

Variable		*n*	Mean ± SD
Child age (months)		61	38.06 ± 16.16
Child weight at birth (kg)		60	3.32 ± 0.50
Child length at birth (m)		61	0.50 ± 0.04
Age started solids (months)		59	5.64 ± 1.81
Age stopped breastfeeding (months)		55	16.43 ± 9.76
Age stopped formula (months)		43	8.93 ± 8.87
Age introduced chicken (months)		44	7.67 ± 2.72
Age introduced beef (months)		42	7.79 ± 2.65
Age introduced pork (months)		40	8.28 ± 2.86
Age introduced fish (months)		41	8.73 ± 2.86
Parent 1 Age (years)		61	34.25 ± 4.76
Parent 2 Age (years)		55	36.47 ± 5.8
Number of people in the household		61	4.08 ± 1.02
Number of children in the household		61	2.03 ± 0.84
**Variable**		**Frequency**	**Percent**
Child sex	Female	32	52%
	Male	29	48%
Parent/caregiver sex	Female	58	95%
	Male	3	5%
Parent/caregiver marital status	Married	56	92%
	Divorced	4	6%
	Single, never married	1	2%
Highest level of education of parent/caregiver	High school/GED	1	2%
	Some college	12	20%
	Associate’s degree	5	8%
	Bachelor’s degree	16	26%
	Master’s degree	21	34%
	Doctorate degree	6	10%
Residence location	Farm/rural	8	13%
	Town less than 5000	2	3%
	Town 5000–10,000	5	8%
	Town/city 10,000–50,000	46	76%
Household income	Less than USD 40,000/year	7	11%
	USD 40,000–50,000/year	10	16%
	USD 60,000–79,000/year	15	25%
	USD 80,000/year or more	29	48%
Race	White	43	71%
	Asian	8	13%
	Two or More Races	2	3%
	Not Reported	8	13%
Ethnicity	Hispanic/Latino	3	5%
	Non-Hispanic/Non-Latino	50	82%
	Not Reported	8	13%

**Table 3:** Estimated dietary intakes of and recommended intakes for beef, protein, iron, zinc, and choline at 6–12 months of age.

Variable	Mean ± SD	Minimum	Maximum	RDA or AI
Beef (g)	4.5 ± 8.5	0.0	48.6	-
Protein (g)	26.5 ± 16.7	0.3	84.6	11
Iron (mg)	9.3 ± 8.7	0.0	39.5	11
Zinc (mg)	6.0 ± 5.3	0.0	25.7	3
Choline (mg)	242.1 ±166.6	3.4	1212.0	150 *

(*n* = 61), SD = standard deviation, RDA = Recommended Dietary Allowance, AI = Adequate Intake, * denotes Adequate Intake value.

**Table 4:** Child cognitive scores at 1–5 years of age.

Variable	*n*	Mean ± SD	Minimum	Maximum
Standard	28	102 ± 11	65	115
Fluid	15	41 ± 11	24	61
DCCS	30	50 ± 10	32	70
Flanker	30	48 ± 8	32	67
LSWM	18	42 ± 11	19	57
PCPS	30	32 ±12	19	65
PSM	26	51 ± 12	33	78

*n* = sample size, Standard = Bayley-4 Standard score (children age 12–35 months); NIH toolbox assessments were used for children age 3–5 years and included: Fluid = Fluid Cognition Composite score, DCCS = Dimensional Change Card Sort, Flanker = Flanker Inhibitory Control and Attention, LSWM = List Sorting Working Memory, PCPS = Pattern Comparison Processing Speed, PSM = Picture Sequence Memory.

**Table 5:** Relationship between dietary intake at 6–12 months and cognitive scores at 1–5 years of age.

	Beef (g)			Protein (g)			Iron (mg)			Zinc (mg)			Choline (mg)		
	*n*	r	*p*	*n*	r	*p*	*n*	r	*p*	*n*	r	*p*	*n*	r	*p*
Standard	28	−0.20	0.32	28	0.12	0.54	28	0.05	0.80	28	−0.01	0.96	28	0.12	0.55
Fluid	15	0.13	0.65	15	0.33	0.24	15	0.29	0.29	15	0.36	0.19	15	0.19	0.49
DCCS	30	0.14	0.47	30	0.25	0.18	30	0.09	0.65	30	0.29	0.12	30	0.29	0.12
Flanker	30	0.41	0.02 *	30	0.37	0.05	30	0.28	0.13	30	0.45	0.01 *	30	0.39	0.03 *
LSWM	18	0.19	0.45	18	0.00	0.99	18	0.27	0.28	18	0.25	0.32	18	−0.11	0.67
PCPS	30	0.02	0.90	30	0.14	0.46	30	0.28	0.14	30	0.22	0.24	30	−0.09	0.63
PSM	26	0.34	0.09	26	0.37	0.06	26	0.15	0.47	26	0.32	0.11	26	0.35	0.08

*n* = sample size, r = Spearman rank correlation coefficient, *p* = *p*-value, significance defined as *p* ≤ 0.05, * remained significant after removing outliers, Standard = Bayley-4 Standard score, Fluid = Fluid Cognition Composite Score, DCCS = Dimensional Change Card Sort, Flanker = Flanker Inhibitory Control and Attention, LSWM = List Sorting Working Memory, PCPS = Pattern Comparison Processing Speed, PSM = Picture Sequence Memory.

## Data Availability

The data presented in this study are available on request from the corresponding author.
